# Eye-Sight

**Published:** 1866-08

**Authors:** 


### EYE-SIGHT.

MILTON'S blindness was the result of over-work and dyspepsia.

One of the most eminent American divines has, for some time, been compelled to forego the pleasure of reading, has spent thousands of dollars in vain, and lost years of time, in consequence of getting up several hours before day, and studying by artificial light. His eyes will never get well.

Multitudes of men and women have made their eyes weak for life, by the too free use of the eyesight in reading small print, and doing fine sewing. In view of these things, it is well to observe the following rules in the use of the eyes.

Avoid all sudden changes between light and darkness.

Never begin to read, or write, or sew, for several minutes after coming from darkness to a bright light.

Never read by twilight, or moonlight, or of a very cloudy day.

Never read or sew directly in front of the light, or window, or door.

It is best to have the light fall from above, obliquely over the left shoulder.

Never sleep so that, on first awaking, the eyes shall open on the light of a window.

Do not use the eyesight by light so scant, that it requires an effort to discriminate.

Too much light creates a glare, and pains and confuses the sight. The moment you are sensible of an effort to distinguish, that moment cease, and take a walk or ride.

As the sky is blue and the earth green, it would seem that the ceiling should be of a bluish tinge, and the carpet green, and walls of some mellow tint.

The moment you are instinctively prompted to rub the eyes, that moment cease using them.

If the eyelids are glued together on waking up, do not forcibly open them; but apply the saliva with the finger—it is the speediest diluent in the world—then wash eyes and face in warm water.

